# The role of long non-coding RNA (lncRNA) nuclear paraspeckle assembly transcript 1 (NEAT1) in chronic periodontitis progression

**DOI:** 10.1080/21655979.2021.2018387

**Published:** 2022-01-16

**Authors:** Lei Zhang, Hui Lv, Yongxin Cui, Rongji Shi

**Affiliations:** a Department of Stomatology, The Second Hospital, Cheeloo College of Medicine, Shandong University, Jinan City, Shandong Province, China; b Shandong Center for Disease Control and Prevention, Infectious Disease Prevention and Control Institute, Jinan City, Shandong Province, China

**Keywords:** NEAT1, miR-200c-3p, *TRAF6*, chronic periodontitis, in vitro

## Abstract

Long non-coding RNA nuclear paraspeckle assembly transcript 1 (NEAT1) is a novel pro-inflammatory factor in severe human diseases. Since inflammatory plays important roles in periodontitis progression, we aimed to explore the role of NEAT1 in chronic periodontitis (CP) in vitro. We established a periodontitis cell model was established by *Porphyromonas gingivalis* lipopolysaccharide (Pg-LPS)-induced periodontal ligament cells (PDLCs). Quantitative reverse transcription polymerase chain reaction (qRT-PCR) was performed to detect the expression of NEAT1, microRNA (miR)-200c-3p, and tumor necrosis factor receptor-associated factor 6 (*TRAF6*). Cell viability, inflammatory factors, and protein expression of Bcl-2, Bax, and *TRAF6* were analyzed by MTT, enzyme-linked immunosorbent assay, and Western blot. The target relationships among NEAT1, miR-200c-3p, and *TRAF6* were predicted by the StarBase/TargetScan software, and further validated by dual-luciferase reporter assay. In this research, NEAT1 is up-regulated in CP tissues and periodontitis model group. Silencing of NEAT1 and over-expression of miR-200c-3p enhanced cell viability and repressed apoptosis in the periodontitis model group. NEAT1 targets miR-200c-3p, and miR-200c-3p further targets *TRAF6*. MiR-200c-3p inhibitor or over-expression of *TRAF6* reversed the promoting effect of NEAT1 knockdown on cell viability, and the inhibiting effects on inflammatory cytokines and cell apoptosis. Consequently, the silencing of NEAT1 inhibits inflammation and apoptosis via targeting miR-200c-3p/*TRAF6* axis, thereby contributing to alleviate the progression of CP. This finding could provide an underlying target for the treatment of CP.

## Introduction

Chronic periodontitis (CP) is a common dental inflammatory disease [1] primarily caused by *Porphyromonas gingivalis (P. gingivalis)*, and its virulence factor lipopolysaccharide (LPS) is the main pathogenic factor of periodontitis [2,3]. Its pathological features include increased subgingival pathogens and connective tissues injuries surrounding the teeth [4,5]. Inflammation of periodontal ligament cells (PDLCs) is important for periodontal ligament tissues regeneration [6,7]. Therefore, this study places emphasis on the regulatory mechanisms underlying the pathogenesis of periodontitis based on Pg-LPS-induced PDLCs inflammatory damage.

Long non-coding RNAs (lncRNAs) have no protein-coding ability with 200 nucleotides in length [8] and are closely associated with inflammatory-related diseases, such as CP [9,10]. Previous studies have found that over-expression of taurine-up-regulated gene 1 (TUG1) facilitates the proliferation of LPS-induced PDLCs and restrains cell apoptosis [11]. Chen et al. suggested that the up-regulation of lncRNA FGD5-antisense RNA 1 (AS1) in LPS-induced PDLCs could protect against periodontitis [12]. On the other hand, silencing of metastasis-associated lung adenocarcinoma transcript 1 (MALAT1) attenuates the inflammatory response in LPS-induced human gingival fibroblasts (HGFs) [13]. These findings imply that the abnormal expression of lncRNAs influences the progression of CP. lncRNA nuclear paraspeckle assembly transcript 1 (NEAT1) modulates inflammation in several diseases, including asthma [14], chronic obstructive pulmonary disease (COPD) [15], diabetic nephropathy [16], and sepsis [17–19]. Importantly, a study has reported dysregulation of lncRNA NEAT1 in gingival tissues or blood samples of patients with periodontitis in comparison with healthy subjects [20]. Huang et al. indicated that the level of lncRNA NEAT1 was up-regulated in PDLCs subjected to compressive force by qRT-PCR and RNA sequencing assay [21]. However, studies of CP involving NEAT1 are restricted to the level of tissues. Therefore, the mechanism of NEAT1 involved in CP in cellular level needs to be further elucidated.

Some microRNAs (miRs) have anti-inflammation roles in CP. For instance, miR-146a or miR-210 over-expression decreases the secretion of IL-1β and IL-6 in LPS-induced periodontal ligament fibroblasts [22,23]. Additionally, miR-200c-3p is reported to exert an anti-inflammatory role in pre-osteoblasts and HGFs, eventually attenuating the development of periodontitis [24,25]. Notably, it remains unclear whether miR-200c-3p is modulated by NEAT1 to exert its anti-inflammatory function in CP pathogenesis.

Tumor necrosis factor receptor-associated factor (*TRAF*) is an oncogene in the pathogenesis of several human cancers, including colorectal [26], gastric [27], breast [28], and prostate [29] cancers. In recent years, the pro-inflammatory role of *TRAF6* has attracted increasing attention [30,31]. *TRAF6* is regulated by lncRNA MIAT aggravates the inflammatory response in LPS-induced septic cardiomyopathy [30]. Silencing of *TRAF6* has a nephroprotective effect on LPS-induced acute renal injury by suppressing inflammation [31]. P22077 could inhibit inflammation and reduce the lung injury by promoting *TRAF6* degradation in LPS-induced endotoxemia mice [32]. The down-regulation of *TRAF6* has a suppressive effect on inflammation in PDLCs induced by *P. gingivalis* [33]. Furthermore, *TRAF6* is also negatively regulated by miR-146a in CP [22]. Nevertheless, the interactions among *TRAF6*, NEAT1, and miR-200c-3p axis in the pathogenesis of CP are relatively unknown.

In this research, we employed CP tissues and LPS-induced PDLCs in vitro to determine whether NEAT1 is involved in the regulation of CP and what is the underlying mechanism in the progression of CP. The result showed that NEAT1 was up-regulated in CP tissues and model group. The silencing of NEAT1 could protect the PDLCs against LPS-induced inflammation and apoptosis by targeting miR-200c-3p/*TRAF6* axis, thereby contributing to alleviate the progression of CP. The results may provide a novel insight for the pathophysiology mechanism of CP and may provide support for NEAT1 in the clinical applications of CP therapy.

## Materials and methods

### Tissues collection

In total, 28 patients with CP without other diseases were selected from 2017 to 2018 in our hospital. Simultaneously, 20 healthy volunteers undergoing a physical examination were recruited. Gingiva tissues were obtained through operation from CP patients, followed by an original ineffectual nonsurgical scaling and root planning in accordance with the established professional and required oral hygiene of patients. Gingival tissues were also procured through crown-lengthening procedures from healthy individuals with the following inclusion criteria: clinical attachment loss <4 mm, probing depth (PD) <4 mm, and no alveolar bone destruction at radiographic level [12]. All the collected gingival tissue samples were frozen in liquid nitrogen and then stored immediately at −80°C for further experiments. The procedures were conducted based on the Declaration of Helsinki and obtained the approval of our hospital’s ethics committee. Each participant has obtained informed consent.

### Isolation, culture, and transfection of human PDLCs

PDLCs were isolated from the healthy periodontal ligament in the middle third of the periodontal ligament root of the third molars as previously described [34]. The cells were cultured in Dulbecco’s Modified of Eagle’s Medium (DMEM). Immunohistochemistry and Alizarin red staining were used to identify the PDLCs as previously described [34]. Immunocytochemistry showed that cells with a spindle shape were positive in vimentin and negative in keratin. Alizarin red staining revealed that the cells could osteogenically differentiate. Consequently, these cells were identified as PDLCs. PDLCs in the third generation were used in the next experiments [35]. PDLCs were then divided into a control group and a model group. PDLCs induced by Pg-LPS (10 μg/mL) were served as the periodontitis model group [36]. These cells served as the control (without LPS treatment). shRNA-negative control (sh-NC), sh-NEAT1, miR-200c-3p inhibitor, miR-200c-3p mimics, miR-NC, and over-expression *TRAF6* vector (pcDNA-*TRAF6*) were co-transfected with LPS-induced PDLCs for 48 h. PDLCs were collected to perform further experiments.

### Quantitative real-time PCR qRT-PCR

The total RNA from PDLCs was extracted using RNAiso Plus (Takara, Tokyo, Japan). RNA was reversed transcribed into cDNA using M-MLV Reverse Transcriptase kit (Sigma-Aldrich). qRT-PCR was performed using a SYBR® Green PCR Kit (Sigma-Aldrich) according to the instructions of the manufacturer. GAPDH or U6 was used for the normalization of the mRNA expression of NEAT1, *TRAF6,* and miR-200c-3p.

### Western blotting assay

The protein samples were extracted using RIPA lysis buffer (Solarbio, Beijing, China). The proteins were then separated by 10% SDS-PAGE and then transferred onto polyvinylidene difluoride (PVDF). The membranes were blocked by 5% skim milk at 25°C for 1 h. Afterward, the membranes were incubated with primary antibodies, including TRAF6 (1:1000, Abcam), Bax (1:1000, Abcam), Bcl-2 (1:1000, Abcam), and β-actin (1:1000, Abcam) at 4°C overnight. Afterward, the membranes were incubated with horseradish peroxidase (HRP)-conjugated secondary antibody at 25°C for 1 h. Blots were visualized and analyzed using a chemiluminescence system (Bio-Rad, CA, USA). β-actin was employed as a protein loading control.

### 3-(4,5-Dimethylthiazol-2-yl)-2,5-diphenyl tetrazolium bromide (MTT) assay

The LPS-induced PDLCs were seeded in 96-well plate and incubated for 24 h. Thereafter, 5 mg/mL of MTT was added to incubate for another 2 h at 37°C with 5% CO_2_. Dimethyl sulfoxide was added to terminate reactions. The viability was analyzed by a microplate reader (Thermo Fisher Scientific) at OD 450.

### Enzyme-linked immunosorbent assay (ELISA)

Culture media was collected from each group, including the control, Model, Model + sh-NEAT1/NC, Model + miR-200c-3p mimics/NC, Model + sh-NEAT1 + miR-200c-3p inhibitor, and Model + sh-NEAT1 + pcDNA-*TRAF6*. The levels of tumor necrosis factor (TNF)-α, interleukin (IL)-6, and IL-1β were detected by ELISA kits according to the manufacturer’s guidelines (Eusebio, Shanghai, China). The absorbance was detected at 450 nm by a microplate reader (Bio-Rad).

### Dual luciferase reporter (DLR) assay

Target-prediction analyses for the targets miRNAs of NEAT1 and miRNA targets sites were formed using TargetScan (http://www.targetscan.org//) and StarBase (https://starbase.sysu.edu.cn/index.php). The 3’-UTR fragment of NEAT1 or *TRAF6* which contained binding sites of miR-200c-3p was introduced into a pGL3-promotor vector to construct NEAT1 wt (mut) or *TRAF6* wt (mut). Next, the above reporter vectors along with miR-NC/miR-200c-3p mimics were transfected into PDLCs via Lipofectamine 3000 (Invitrogen) according to the manufacturer’s instructions. After transfection for 48 h, relative luciferase activity was assessed with a Dual-Glo Luciferase assay kit (Promega, Madison, WI, USA).

### Statistical analysis

Data were expressed as the mean ± SD and analyzed using SPSS 20.0 (SPSS; Chicago, USA). Student’s t-test was used to analyze the differences between the two groups. One-way ANOVA followed by Tukey post-hoc tests and two-way ANOVA was used for comparisons between two groups or among multiple groups. *P*< 0.05 was considered to be a statistically significant difference.

## Results

In this study, we established a periodontitis cell model by Pg-LPS-induced PDLCs. We determined the levels of TNF-α, IL-1β, and IL-6, as well as protein expression of Bcl-2, Bax, and TLR4 by ELISA and Western blot. The expression of NEAT1, miR-200c-3p, and TRAF6 was detected by qRT-PCR. The results showed that the silencing of NEAT1 could protect the PDLCs against LPS-induced inflammation and apoptosis by targeting miR-200c-3p/*TRAF6* axis, thereby contributing to alleviate the progression of CP.

### Knockdown of NEAT1 inhibits inflammatory responses in model group

**Figure 1.**
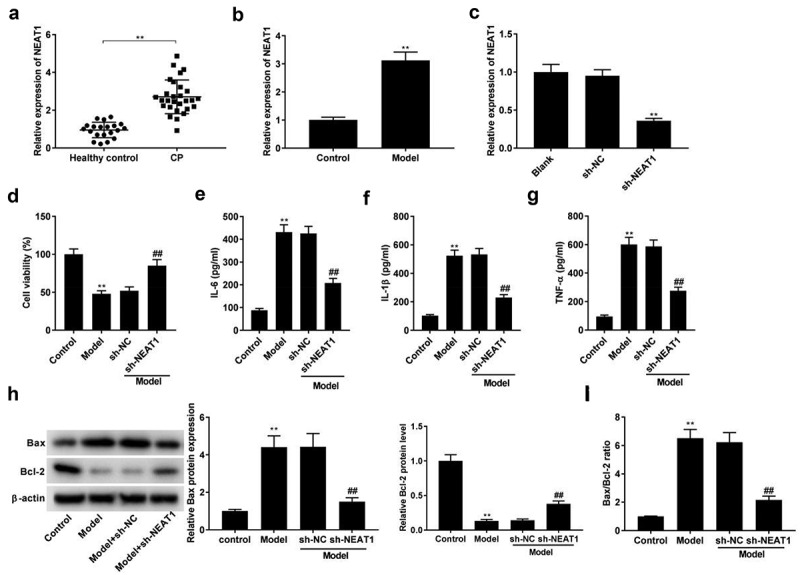
Knockdown of NEAT1 inhibits inflammation in model group. (a) NEAT1 expression in healthy control individuals and CP tissues. **P < 0.01 vs. healthy control. (b) NEAT1 expression in model group. **P< 0.01 vs. Control. (c) The transfection efficiency of NEAT1 was detected by qRT-PCR. **P< 0.01 vs. sh-NC. (d-g) Cell viability, and the levels of IL-6, IL-1β, and TNF-α in control, model, model + sh-NC, and model + sh-NEAT1 groups. **P< 0.01 vs. Control. ^##^*P* < 0.01 vs. model + sh-NC. (h) Relative protein expression of Bax and Bcl-2 was detected by Western blot. **P< 0.01 vs. Control. ^##^*P* < 0.01 vs. model + sh-NC. (i) The ratio of Bax/Bcl-2 in control, model, model + sh-NC, and model + sh-NEAT1 groups. **P< 0.01 vs. Control. ^##^*P* < 0.01 vs. model + sh-NC.

The expression of NEAT1 was initially determined by qRT-PCR, and we found that NEAT1 was highly expressed in CP tissues (Figure 1a, *p* < 0.01) and the model group compared with respective controls (Figure 1b, *p* < 0.01). qRT-PCR assay showed that the expression of NEAT1 was decreased in sh-NEAT1 group compared with sh-NC group (Figure 1c, *p* < 0.01), suggesting the successful transfection of sh-NEAT1. The viability of PDLCs was decreased after LPS stimulation, while sh-NEAT1 reversed the inhibitory effect of LPS on cell viability (Figure 1d, *p* < 0.01). Thereafter, the influences of NEAT1 silencing on inflammatory responses were researched. As illustrated in Figure 1e-g, the levels of IL-6, IL-1β, and TNF-α were distinctly promoted in model groups. However, the promoting roles of LPS in these inflammatory cytokines were reversed by sh-NEAT1 (*P* < 0.01). Western blotting assay uncovered that the LPS treatment had a promoting effect on Bax level, and a suppressive effect on Bcl-2 protein expression (Figure 1h, *p* < 0.01). As expected, the transfection of sh-NEAT1 significantly reversed the effects of LPS on Bax and Bcl-2 (Figure 1h, *p* < 0.01). LPS was also found to dramatically elevate the Bax/Bcl-2 ratio, which was reversed by the transfection of sh-NEAT1 (Figure 1i, *p* < 0.01).

### NEAT1 targets miR-200c-3p

**Figure 2.**
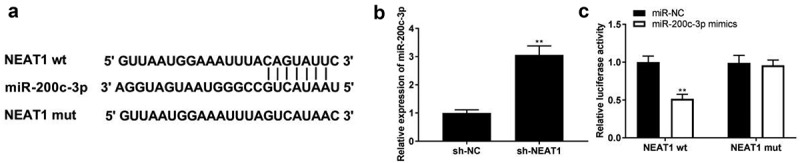
NEAT1 targets miR-200c-3p. (a) The binding site of NEAT1 and miR-200c-3p was predicted by TargetScan. (b) The expression of miR-200c-3p was detected by qRT-PCR. **P < 0.01 vs. sh-NC. (c) The interaction between NEAT1 and miR-200c-3p was determined by DLR assay. **P < 0.01 vs. miR-NC.

StarBase was used to predict the relationship between NEAT1 and miR-200c-3p, and the binding site is shown in Figure 2a. We found that NEAT1 knockdown elevated miR-200c-3p expression (Figure 2b, *p* < 0.01). In addition, miR-200c-3p mimics co-transfected with NEAT1 wt remarkably declined luciferase activity in PDLCs (Figure 2c, *p* < 0.01), which confirmed that NEAT1 directly targeted miR-200c-3p.

### Over-expression of miR-200c-3p represses inflammation of in model group

**Figure 3.**
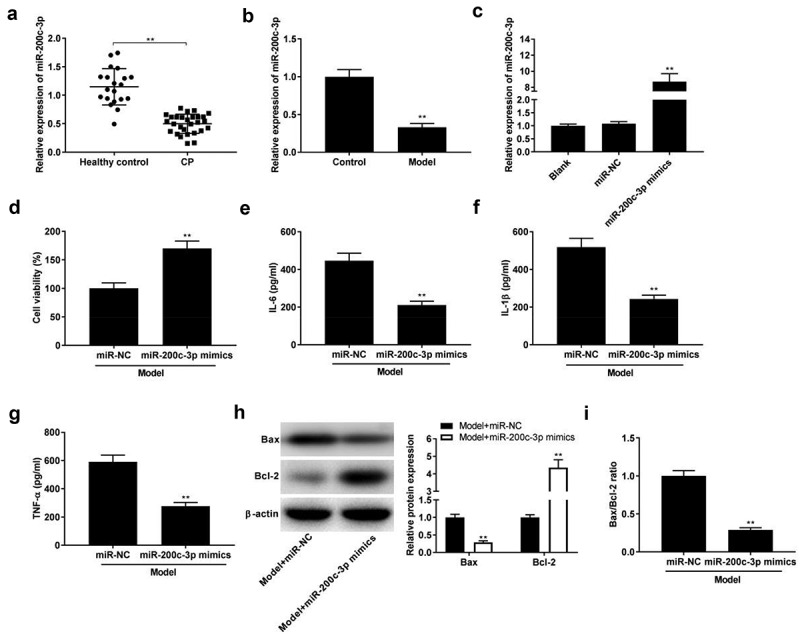
Over-expression of miR-200c-3p inhibits inflammatory response in model group. (a) The expression of miR-200c-3p in healthy control individuals and CP tissues. **P < 0.01 vs. healthy control. (b) The expression of miR-200c-3p in model group. **P< 0.01 vs. control. (c) The expression of miR-200c-3p was detected by qRT-PCR after transfection of miR-NC and miR-200c-3p mimics. **P < 0.01 vs. miR-NC. (d-g) Cell viability and the levels of IL-6, IL-1β, and TNF-α in model + miR-NC and model + miR-200c-3p mimics groups. **P< 0.01 vs. miR-NC. (h-i) Relative protein expression of Bax and Bcl-2, and the ratio of Bax/Bcl-2 in model + miR-NC and model + miR-200c-3p mimics groups. **P < 0.01 vs. miR-NC.

In order to explore the function of miR-200c-3p in CP, the expression of miR-200c-3p was detected by qRT-PCR, and we found that miR-200c-3p was down-expressed in CP tissues and model group (Figure 3a-b, *P* < 0.01). Then, miR-200c-3p mimics were successfully transfected into model group to explore the function of miR-200c-3p on CP progression in vitro (Figure 3c, *p* < 0.01). As presented in Figure 3d-g, miR-200c-3p mimics remarkably facilitated the viability (*P* < 0.01) and repressed the levels of IL-6, IL-1β, and TNF-α (*P*< 0.01). In addition, miR-200c-3p mimics down-regulated the expression of Bax and up-regulated Bcl-2 expression, as well as decreased Bax/Bcl-2 ratio (Figure 3h-i, *P* < 0.01).

### TRAF6 is a target of miR-200c-3p

**Figure 4.**
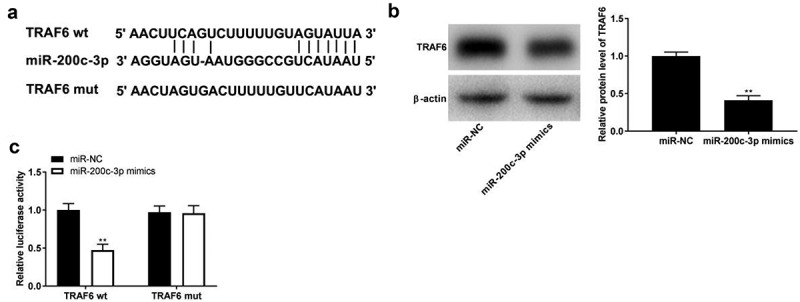
*TRAF6* is a target of miR-200c-3p. (a) The binding sites of *TRAF6* and miR-200c-3p were predicted by StarBase software. (b) The expression of *TRAF6* was detected by Western blot. **P < 0.01 vs. miR-NC. (c) Target relationship of *TRAF6* and miR-200c-3p. **P < 0.01 vs. miR-NC.

The binding site between miR-200c-3p and *TRAF6* was predicted using TargetScan software, and a binding site is shown in Figure 4a. Western blot assay showed that *TRAF6* expression was significantly decreased by the transfection of miR-200c-3p mimics (Figure 4b, *p*< 0.01). The DLR assay indicated that luciferase activity significantly decreased in PDLCs co-transfected with *TRAF6*-wt and miR-200c-3p mimics by contrast to in the *TRAF6*-wt + miR-NC group (Figure 4c, *p*< 0.01).

### Silencing of NEAT1 inhibits inflammation and apoptosis by targeting miR-200c-3p/TRAF6 axis

**Figure 5.**
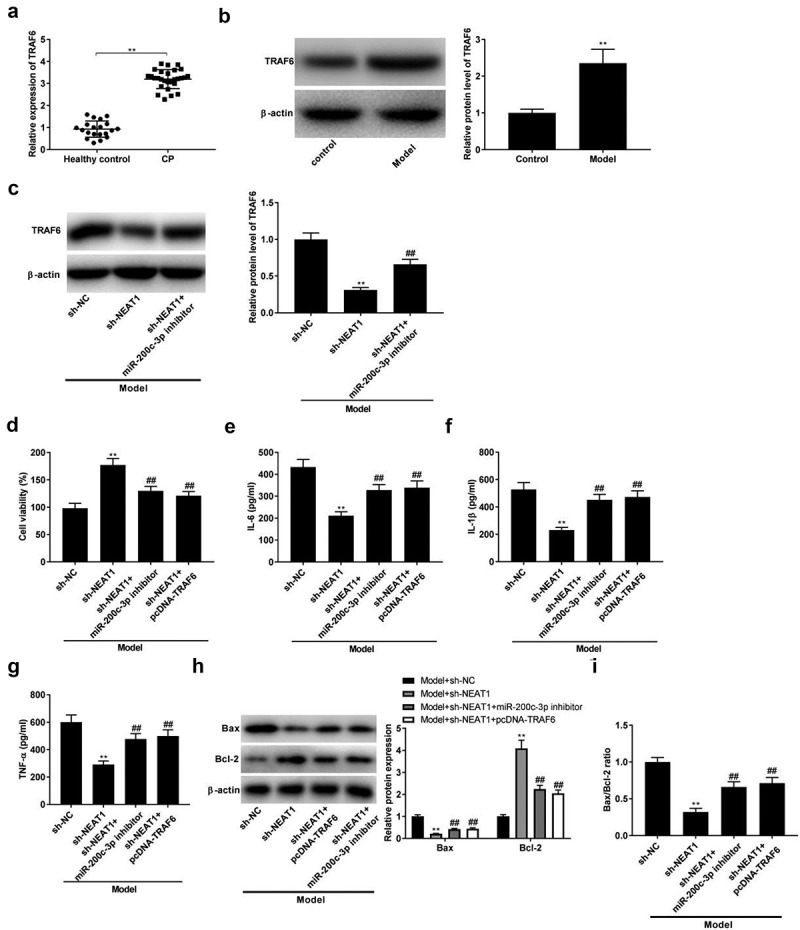
Silencing of NEAT1 inhibits inflammation and apoptosis by targeting miR-200c-3p/*TRAF6* axis. (a) *TRAF6* expression in healthy control individuals and CP tissues. **P < 0.01 vs. healthy control. (b) *TRAF6* expression in control and model groups. **P< 0.01 vs. control. (c) The expression of *TRAF6* was determined by Western blot after transfection of sh-NEAT1/sh-NEAT1 + miR-200c-3p inhibitor. **P < 0.01 vs. sh-NC. ^##^*P* < 0.01, vs. sh-NEAT1. (d-g) Cell viability and the levels of IL-6, IL-1β, and TNF-α in model + sh-NC, model + sh-NEAT1, model + sh-NEAT1 + miR-200c-3p inhibitor, and model + sh-NEAT1 + pcDNA-*TRAF6* groups. **P < 0.01 vs. sh-NC. ^##^*P* < 0.01, vs. sh-NEAT1. (h-i) Relative protein expression of Bax and Bcl-2, and the ratio of Bax/Bcl-2 in Model + sh-NC, model + sh-NEAT1, model + sh-NEAT1 + miR-200c-3p inhibitor, and model + sh-NEAT1 + pcDNA-*TRAF6* groups. **P < 0.01 vs. sh-NC. ^##^*P* < 0.01, vs. sh-NEAT1.

The expression of *TRAF6* in CP tissues and model group was visibly up-regulated compared with respective controls (Figure 5a-b, *P* < 0.01). At the same time, we analyzed the protein level of *TRAF6* in model group. The results showed that protein expression of *TRAF6* was down-regulated by sh-NEAT1, while miR-200c-3p inhibitor partly reversed the effect of sh-NEAT1 on protein expression of *TRAF6* in model group (Figure 5c, *p* < 0.01). Subsequently, feedback verification experiments were performed to investigate the interactions among NEAT1, miR-200c-3p, and *TRAF6* on CP progression in vitro. As illustrated in Figure 5d, the transfection of miR-200-3p inhibitor and pcDNA-*TRAF6* reversed the promoting effect of sh-NEAT1 on viability in model groups (Figure 5d, *p* < 0.01). The anti-inflammatory effects of sh-NEATT were markedly reversed by the transfection of miR-200-3p inhibitor and pcDNA-*TRAF6* in model groups (Figure 5e-g, *P* < 0.01). Moreover, miR-200-3p inhibitor and pcDNA-*TRAF6* also reversed the effects of sh-NEATT on the expression of Bcl-2 and Bax, and the ratio of Bax/Bcl-2 in model groups (Figure 5h-i, *P* < 0.01).

## Discussion

A study showed that Pg-LPS inhibited cell viability and triggered inflammation of PDLCs [36]. Consistent with this study, our result showed that LPS stimulation reduced the viability of PDLCs and increased the levels of inflammatory factors (IL-6, IL-1β, and TNF-α) in model groups. Furthermore, LncRNA NEAT1 plays a crucial role in several inflammation diseases [14,16,18,37]. The up-regulation of NEAT1 occurs in the tissues of patients with asthma, COPD, and acute kidney injury [14,16,38]. In this study, we found that NEAT1 was significantly up-expressed in CP tissues. Consistent with our results, Sayad et al. also indicated that NEAT1 expression in CP tissues is dramatically elevated [20]. Additionally, we showed that the expression of NEAT1 was up-regulated in model group, suggesting that NEAT1 may play a vital role in CP. To further explore the exact role of NEAT1 in CP, relevant in vitro experiments were performed in model groups. We demonstrated that the suppression of NEAT1 facilitates viability and inhibits apoptosis and inflammation in model group. In line with these results, Yi et al. discovered that knockdown of NEAT1 could promote cell viability, and suppress inflammatory factors and cell apoptosis in sepsis-induced acute kidney injury [38]. Therefore, we speculated that the silencing of NEAT1 might be a suppressor in the occurrence and development of CP.

miRNAs act as suppressors to participate in the inflammation reaction of CP in vitro or in vivo [23,39,40]. A decreased expression of miR-335-5p has been found in periodontitis tissues [40], and miR-210 is low-expressed not only in CP tissues but also in model group [23]. Additionally, miR-218 is minimally expressed both in CP tissues and periodontal ligament progenitor cells [39]. In this study, miR-200c-3p was reduced in CP tissues and PDLCs, and over-expression of miR-200c-3p reduced the levels of inflammatory factors in model group, which was consistent with previous studies [24,25]. Additionally, in the present study, we demonstrated that the up-regulation of miR-200c-3p promoted cell viability and inhibited apoptosis in model group. More importantly, miR-200c-3p was found to be up-regulated by sh-NEAT1, and a target of NEAT1. Our feedback verification experiments suggested that inhibition of miR-200c-3p reversed the effects of sh-NEAT1 on cell viability, cell apoptosis, and inflammatory factors in PDLCs. The above data indicated that silencing of NEAT1 inhibited apoptosis and inflammation model groups by up-regulating miR-200c-3p expression, thereby contributing to alleviate the progression of CP.

*TRAF6* is involved in the regulation of CP [33,41] and is highly expressed in LPS-induced PDLCs [41]. LPS stimulation elevates *TRAF6* expression in PDLCs [33]. Similarly, we found that the protein level of *TRAF6* was up-regulated in the model group, and an increased expression of *TRAF6* in CP tissues was observed. The result indicated that *TRAF6* may be closely related to the progression of CP. Simultaneously, we verified that *TRAF6* was a target gene of miR-200c-3p and silencing of NEAT1 down-regulated *TRAF6* expression. The results of the feedback verification experiment verified that the over-expression of *TRAF6* reversed the anti-inflammation and anti-apoptosis effects, as well as the promoting effect on the cell viability of sh-NEAT1. In other words, we believed that silencing NEAT1 alleviated the progression of CP via regulation of the miR-200c-3p/*TRAF6* axis.

## Conclusions

In conclusion, NEAT1 was significantly highly expressed in CP tissues and LPS-induced PDLCs. Silencing of NEAT1 markedly suppressed the inflammatory response and apoptosis via the miR-200c-3p/*TRAF6* axis in the model group. Accordingly, NEAT may act as a potential therapeutic target for CP therapy in clinical applications. In addition, we failed to verify the NEAT1/miR-200c-3p/TRAF6 axis in vivo, and this may be a limitation of this study. We will elucidate this in the future.

## Highlights

1. NEAT1 is up-regulated in CP tissues and model group.
2. NEAT1 targets miR-200c-3p in model group.
3. *TRAF6* is a target of miR-200c-3p.
4. Silencing of NEAT1 inhibits inflammation and apoptosis by targeting miR-200c-3p/*TRAF6* axis.
